# Intradural cauda equina hemangioblastomas: Surgical management and long-term outcomes at a von Hippel–Lindau referral center

**DOI:** 10.1016/j.bas.2026.107479

**Published:** 2026-10-12

**Authors:** Roberto Doria-Medina, Nicolas Neidert, Marc Hohenhaus, Christine Julia Gizaw, Kilian Rhein, Athina Ganner, Romina Hohenhaus, Jürgen Beck, Jan-Helge Klingler

**Affiliations:** a Department of Neurosurgery, Medical Center – University of Freiburg, Faculty of Medicine, University of Freiburg, Germany; b Department of Neurosurgery and Spine Surgery, Klinikum Main-Spessart, Lohr am Main, Germany; c Renal Division, Department of Medicine, Medical Center – University of Freiburg, Faculty of Medicine, University of Freiburg, Germany

**Keywords:** Hemangioblastoma, von Hippel–Lindau disease, Cauda equina, Surveillance, Surgical management

## Abstract

**Introduction:**

Spinal hemangioblastomas (sHBLs) are rare vascular tumors occurring sporadically or in association with von Hippel-Lindau (VHL) disease. Intradural cauda equina sHBLs are uncommon and understudied.

**Research question:**

We aimed to describe their surgical treatment and outcomes at a VHL referral center.

**Methods:**

We retrospectively analyzed a prospectively maintained, surgically ascertained VHL/HBL registry of 237 patients who underwent 565 procedures for 804 resected HBLs between January 1997 and July 2025, with follow-up through February 2026. Functional status was assessed using McCormick grade.

**Results:**

Intradural cauda equina sHBLs were identified in 32/237 registry patients (13.5%). Eight patients (25%) underwent eight procedures involving ten tumors; the remaining 24 patients had no resection. Reasons for resection were symptoms and/or radiological progression (n = 7), concomitant removal during surgery for another lesion (n = 2), and for diagnostics due to initial presentation (n = 1). All ten tumors were completely resected. At first follow-up, McCormick grade was unchanged in all eight patients, and radicular pain improved in all six symptomatic patients. Median imaging follow-up was 67 months (range, 11–260), with no recurrence was identified. One postoperative cerebrospinal fluid fistula resolved after lumbar drainage. One patient experienced a clinical deterioration after 200 months due to VHL disease burden.

**Discussion and conclusion:**

Cauda equina sHBLs were infrequently resected in this selected registry. At our center, management is individualized within a structured surveillance program. Surgical decisions are guided by symptoms, radiological progression, lesion location, and clinical context, with favorable outcome. Long-term surveillance at an experienced VHL center remains essential.

## Highlights

• Cauda equina sHBLs were identified in 13.5% of registry patients.
• Eight of 32 affected patients underwent cauda equina resection.
• Symptoms or concerning radiological progression prompted most tumor resections.
• All ten operated tumors were completely resected.
• No recurrence was identified during long-term imaging follow-up.

## 1 Introduction

Hemangioblastomas (HBLs) are rare, highly vascular tumors of the central nervous system (CNS), most commonly occurring in the cerebellum, brainstem, and spinal cord (Gläsker, 2005). They may arise sporadically or in association with von Hippel-Lindau (VHL) disease, an autosomal dominant tumor-predisposition syndrome caused by pathogenic variants in the VHL tumor suppressor gene (Kaelin, 2002; Lonser et al., 2003). VHL disease predisposes affected individuals to multiple HBLs, often beginning in early adulthood and frequently involving the spinal cord (Takami et al., 2022). Surgically treated spinal hemangioblastomas (sHBLs) are most commonly intramedullary and may cause progressive neurological deficits through tumor mass effect, cyst formation, or peritumoral edema (Wanebo et al., 2003; Lonser et al., 2014; Wach et al., 2025).

Intradural cauda equina sHBLs constitute a distinct and understudied subgroup (Lonser et al., 2014; Fujii et al., 2022; D’Oria et al., 2022; Han et al., 2024). Although cauda equina sHBLs are frequently detected in VHL surveillance cohorts, surgically treated lesions at this location remain uncommon (Lonser et al., 2014). Consequently, evidence concerning their operative management and long-term outcomes consists primarily of case reports and small surgical series (D’Oria et al., 2022; Han et al., 2024; Mehta et al., 2017).

Because cauda equina sHBLs compress individual nerve roots or fascicles rather than spinal cord parenchyma, their clinical presentation and treatment considerations may differ from those of intramedullary tumors. These lesions may attain substantial size before causing symptoms such as radicular pain, sensory disturbance, or motor deficit (Lonser et al., 2014). This anatomical distinction may permit observation of selected asymptomatic lesions; however, whether prolonged surveillance can be undertaken without jeopardizing neurological function remains uncertain. Lesions close to the conus medullaris may warrant particular attention because further growth could place functionally important neural structures at risk.

As a specialized interdisciplinary VHL referral center caring for more than 300 patients with VHL disease patients annually, we maintain a registry of patients who have undergone surgery for CNS HBLs (Klingler et al., 2020). We therefore aimed to characterize the clinical and imaging presentation, indications for surgery, surgical management, and postoperative functional and radiological outcomes of intradural cauda equina sHBLs. In addition, we sought to describe their observed management within a surgically ascertained referral-center registry.

## 2 Materials and methods

### 2.1 Study design and setting

We conducted a retrospective analysis of a prospectively maintained institutional VHL/HBL registry (Klingler et al., 2020), implemented using the Research Electronic Data Capture (REDCap) platform. The registry includes patients who underwent surgery for at least one CNS HBL at our center. Clinical and imaging data were entered by the VHL team during routine outpatient surveillance visits and hospital admissions.

We screened all 237 patients registered between January 1, 1997, and July 31, 2025, by reviewing the available clinical records and imaging, irrespective of whether an identified cauda equina lesion had been resected. Evaluable imaging of the cauda equina was available for all registry patients. The surgical ascertainment cutoff was July 31, 2025; follow-up data for patients operated on by that date were updated through February 26, 2026.

Cauda equina sHBLs were defined as intradural extramedullary HBLs arising along the cauda equina below the conus medullaris. Intramedullary, mixed intra-/extradural, and foraminal nerve-root lesions were excluded. A surgical procedure remained eligible if it included resection of an intradural cauda equina sHBL, even when a separate HBL in another anatomical compartment was resected during the same procedure.

Patients without resection of a cauda equina sHBL were included only to describe lesion ascertainment and observed management within the registry. Their clinical characteristics, radiological course, and outcomes were not comparatively analyzed.

### 2.2 Data collection

At the patient level, we recorded VHL status and whether cauda equina resection had been performed at our institution by the surgical ascertainment cutoff. For patients who underwent surgery, the collected variables included age at surgery, sex as documented in the medical record, family history of VHL disease, body mass index (BMI), American Society of Anesthesiologists (ASA) class, postoperative length of stay (LOS), operative duration, and estimated blood loss (EBL).

Preoperative and postoperative motor deficits, sensory deficits, radicular pain, gait disturbance, and bladder dysfunction were recorded. Functional status was assessed using the McCormick grade (McCormick et al., 1990) preoperatively, at the first recorded postoperative follow-up, and at the last available clinical follow-up.

Tumor-level variables included anatomical level, maximum orthogonal diameters, calculated volume, presence of a cystic component, duration of radiological observation before resection, extent of resection, and recurrence during available postoperative imaging follow-up. Tumor volume was estimated using formula A × B × C/2, where A, B, and C represented the maximum orthogonal tumor diameters.

Reasons for resection were abstracted from the clinical records and categorized as symptoms and/or radiological progression, concomitant removal during surgery for another lesion, or diagnostic clarification at initial presentation. Surgical variables included the operative approach, number of surgical exposures, retractor system, use of preoperative embolization, use of intraoperative neuromonitoring (IONM), dural-closure technique, intraoperative blood transfusion, and postoperative complications.

### 2.3 Outcomes and follow-up

Outcomes of interest included McCormick grade at the first recorded postoperative follow-up, change in radicular pain, extent of resection, postoperative complications, and tumor recurrence during available imaging follow-up. Long-term clinical changes and surgery for postoperative spinal instability were also recorded.

Patients with VHL disease were invited to participate in annual multidisciplinary VHL-specific surveillance, including contrast-enhanced T1-weighted magnetic resonance imaging (MRI) of the entire neuraxis. Postoperative MRI was scheduled approximately 3 months after surgery and annually thereafter. The first postoperative MRI was reviewed for residual tumor, and subsequent examinations were reviewed for recurrence.

### 2.4 Statistical analysis

Data were analyzed descriptively. Continuous variables were summarized as median and range or as mean and standard deviation as appropriate. Categorical variables were reported as counts and percentages. Denominators were specified according to the relevant unit of analysis, including patients, procedures, surgical exposures, or tumors. No inferential statistical analyses were performed because of the small cohort size and descriptive study design.

### 2.5 Ethical approval

This study was performed in line with the principles of the Declaration of Helsinki. Approval was granted by the Ethics Committee of University of Freiburg (No. 79/20). Informed consent was obtained from all individual participants for inclusion in the VHL/HBL registry.

## 3 Results

### 3.1 Screening of the institutional VHL/HBL registry

As of July 31, 2025, the institutional VHL/HBL registry comprised 237 patients who had undergone 565 procedures involving 804 resected CNS HBLs. Out of 565 surgeries performed, 533 cases (94.3%) were intracranial or intramedullary HBLs. Among the remaining 32 procedures, 15 involved spinal extramedullary lesions at or above the conus medullaris, and nine involved extradural lesions below the conus medullaris. The final surgical cohort comprised eight patients who underwent eight procedures involving ten intradural cauda equina sHBLs. Procedures remained eligible when they included an intradural cauda equina sHBL, even if a separate HBL in another anatomical compartment was resected during the same procedure. The eight procedures, ten tumors, and eight patients represented 1.4% (8/565) of all procedures, 1.2% (10/804) of all resected CNS HBLs, and 3.4% (8/237) of registry patients, respectively (Fig. 1).

**Fig. 1:**
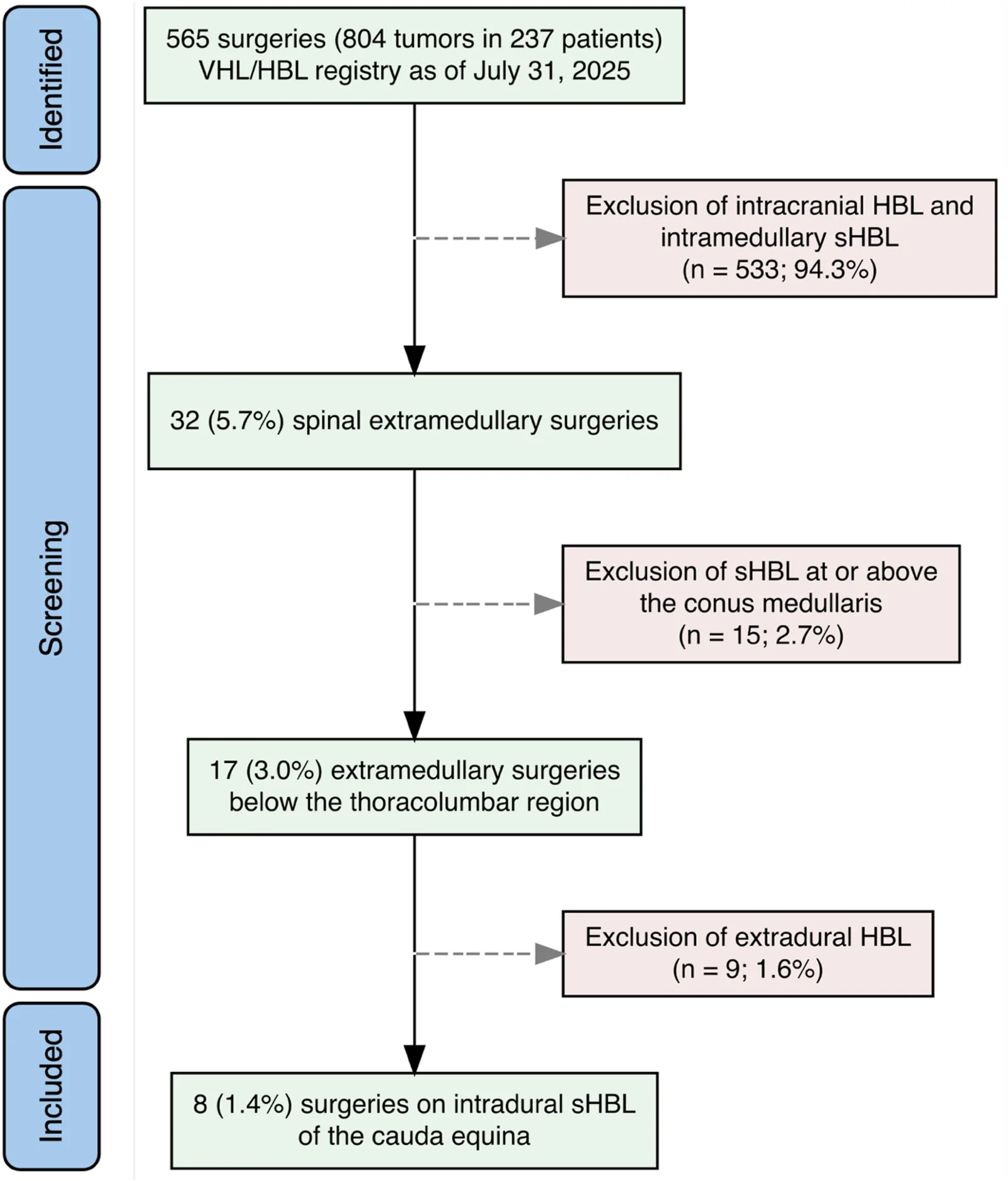
Procedure-level selection of the intradural cauda equina surgical cohort from the institutional VHL/HBL registry. Between January 1, 1997, and July 31, 2025, 237 registry patients underwent 565 procedures involving 804 resected CNS HBLs. Thirty-two procedures involved at least one spinal extramedullary HBL, and 17 involved at least one spinal extramedullary lesion below the conus medullaris. After exclusion of nine procedures involving an extradural below-conus lesion without an eligible intradural cauda equina HBL, the final cohort comprised eight procedures in eight patients involving ten intradural cauda equina sHBLs, representing 1.4% of all surgeries, 1.2% of all resected CNS HBLs, and 3.4% of all patients in our registry, respectively. Abbreviations: HBL, hemangioblastoma; sHBL, spinal hemangioblastoma; VHL, von Hippel-Lindau.

At the patient level, review of the available records and imaging identified an intradural cauda equina sHBL in 32 of 237 registry patients (13.5%). Of these 32 patients, 31 had VHL disease and one had a sporadic lesion. By July 31, 2025, eight patients (25%) had undergone cauda equina resection at our institution. The remaining 24 patients had no cauda equina resection recorded at our institution by that date.

### 3.2 Preoperative characteristics

Among the eight patients who underwent surgery, the median age was 48 years (range, 25–62), and the mean BMI was 25.3 ± 5.8 kg/m^2^. Six patients were classified as ASA class II and two as ASA class III. Four patients were female. Seven patients had VHL disease, and one had a sporadic sHBL.

Six patients (75%) were symptomatic before surgery. All six reported radicular pain corresponding to the affected nerve root. Three patients had combined motor and sensory deficits, and two had an isolated motor deficit. No patient had gait disturbance or bladder dysfunction. Preoperative McCormick grade was I in five patients (62.5%) and II in three patients (37.5%).

At the tumor level, seven lesions were resected because of attributable symptoms and/or radiological progression, two were removed concomitantly during surgery for another lesion, and one was resected for diagnostic clarification at the initial presentation of a patient without VHL disease. One lesion adjacent to the conus medullaris at L1/L2 was resected after radiological progression because further growth was considered to pose a risk of irreversible neurological deficit. Of the two concurrently removed tumors, one was resected during surgery primarily undertaken for an intramedullary conus sHBL, and the other during surgery for another cauda equina sHBL.

The median tumor volume was 936 mm^3^ (range, 65–2078). All ten tumors were solid and had no associated cyst. The nine VHL-associated tumors had been followed radiographically for a median of 65 months (range, 31–178) before resection. Three of these tumors were first identified during annual surveillance, whereas six were visible on the first available MRI. The preoperative characteristics are summarized in Table 1, and the anatomical distribution of the ten resected tumors is shown in Fig. 2.

**Table 1:** Preoperative patient and tumor characteristics and indication for resection.

**Patient characteristics (n = 8)**	
Age at surgery, median (range), years	48 (25–62)
Body mass index, mean (SD), kg/m^2^	25.3 (5.8)
**ASA physical status, n (%)**	
II	6 (75)
III	2 (25)
**Sex, n (%)**	
Female	4 (50)
Male	4 (50)
VHL disease, n (%)	7 (87.5)
Sporadic disease, n (%)	1 (12.5)
**Preoperative clinical findings, n (%)**^a^	
Radicular pain	6 (75)
Motor deficit	6 (75)
Sensory deficit	3 (37.5)
Gait disturbance	0 (0.0)
Bladder dysfunction	0 (0.0)
**Preoperative McCormick grade, n (%)**	
Grade I	5 (62.5)
Grade II	3 (37.5)
**Tumor volume, median (range), mm^3^ (n = 10)**	936 (65–2078)
**Reason for resection, n (%)**^b^	
Symptoms and/or radiological progression	7 (70.0)
Concomitant resection during a procedure for another lesion	2 (20.0)
Diagnostic clarification at initial presentation	1 (10.0)
Preoperative radiological observation of VHL-associated tumors, median (range), months^c^	65 (31–178)

Data are presented as n (%) unless otherwise stated.

Abbreviations: ASA, American Society of Anesthesiologists; SD, standard deviation; VHL, von Hippel-Lindau.

a Clinical findings were not mutually exclusive. Percentages are based on all eight patients.

b Reasons for resection are reported per tumor (n = 10). Symptoms and radiological progression were not mutually exclusive within the combined category.

c Seven patients with VHL disease contributed nine VHL-associated tumors. Preoperative radiological observation was calculated for these nine tumors.

**Fig. 2:**
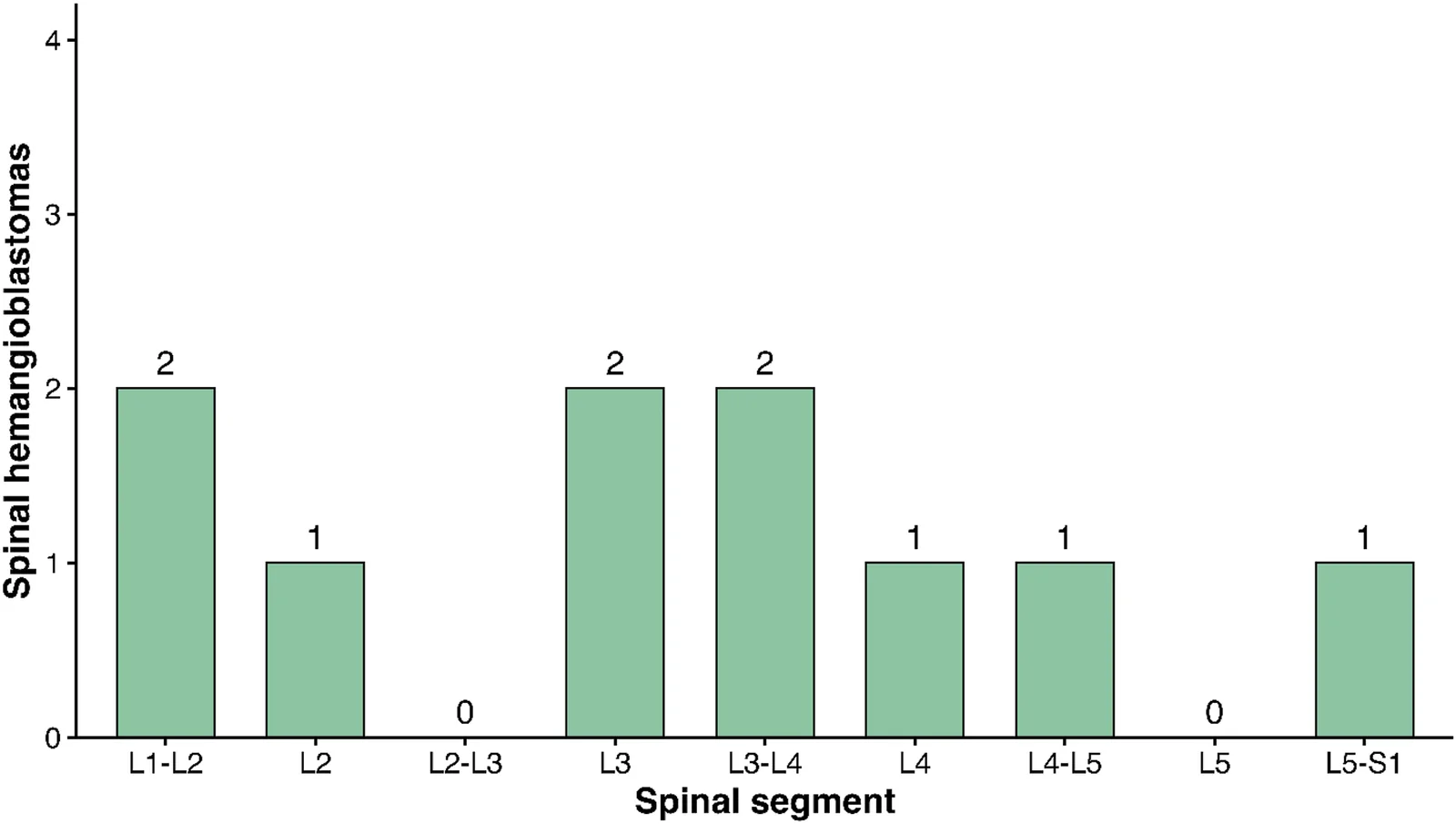
Distribution of spinal segments of ten resected intradural cauda equina hemangioblastomas. Anatomical distribution of ten resected intradural spinal hemangioblastomas of the cauda equina (n = 10). Bars represent the proportion of tumors located at each lumbar or lumbosacral segment level, expressed as a percentage of all tumors.

### 3.3 Surgical characteristics

The median operative duration was 228 min (range, 147–400), and the median EBL was 170 mL (range, 100–500). No patient received an intraoperative blood transfusion. IONM was used in three procedures, and preoperative embolization was performed before one procedure.

Two procedures involved resection of two cauda equina sHBLs. One of these procedures involved tumors at different spinal levels, whereas the other involved two distinct tumors at the same level. One procedure also included resection of an intramedullary conus HBL, which was the primary target of that operation.

Because one procedure required separate exposures at two spinal levels, nine surgical exposures were performed across the eight procedures. The recorded approaches comprised four hemilaminectomies (44.4%), three laminotomies (33.3%), one partial hemilaminotomy (11.1%), and one facetectomy (11.1%). Traditional retractors were used for eight exposures (88.9%), and a minimally invasive tubular retractor was used for one (11.1%) (Krüger et al., 2019). The dura was closed using sutures alone after one exposure (11.1%) and sutures with augmentation after eight (88.9%). Augmentation materials included TachoSil®, Gelita®, fibrin glue, and autologous muscle tissue. Further surgical details are provided in Supplementary Table 1 and Supplementary Fig. 1.

### 3.4 Postoperative outcome and follow-up

The median postoperative LOS was 6 days (range, 4–9). The median interval to the first recorded clinical and imaging follow-up was 3 months (range, 1–20). Complete resection was achieved for all ten tumors.

The median postoperative imaging follow-up was 67 months (range, 11–260), and the median postoperative clinical follow-up was 53 months (range, 3–260). The patient with the sporadic cauda equina sHBL had no long-term clinical or imaging follow-up at our center; however, no residual tumor was identified at the first postoperative assessment.

At the first postoperative follow-up, McCormick grade remained unchanged in all eight patients. Radicular pain improved in all six affected patients. One patient subsequently deteriorated from McCormick grade II to grade III at 200 months in the context of cumulative VHL-associated tumor burden.

One patient developed a postoperative cerebrospinal fluid (CSF) fistula, which was resolved after lumbar drainage without permanent sequelae or deterioration in functional status. No postoperative hematoma, surgical-site infection, unplanned revision surgery, or spinal instability requiring fusion was recorded. No tumor recurrence was identified during the available postoperative imaging follow-up. Postoperative outcomes are summarized in Table 2.

**Table 2:** Postoperative outcome and follow-up of the surgical cohort.

**Patient- and procedure-level outcomes (n = 8)**	
Postoperative length of stay, median (range), days	6 (4–9)
Time to first postoperative clinical and imaging follow-up, median (range), months	3 (1–20)
Postoperative imaging follow-up, median (range), months	67 (11–260)
Postoperative clinical follow-up, median (range), months	53 (3–260)
**McCormick grade at first postoperative follow-up, n (%)**	
Grade I	5 (62.5)
Grade II	3 (37.5)
Unchanged from preoperative grade	8 (100)
Radicular pain improved at first follow-up, n/N (%)	6/6 (100)
Late deterioration in McCormick grade during follow-up, n (%)^a^	1 (12.5)
**Postoperative adverse events, n (%)**	
Cerebrospinal fluid fistula requiring lumbar drainage	1 (12.5)
Hematoma	0 (0.0)
Surgical site infection	0 (0.0)
Spinal instability requiring fusion during available follow-up	0 (0.0)
Unplanned revision surgery	0 (0.0)
**Tumor-level outcomes (n = 10)**	
Complete resection, n (%)	10 (100)
Recurrence during available postoperative imaging follow-up, n (%)^b^	0 (0.0)

Data are presented as n (%) unless otherwise stated. Each patient underwent one procedure.

Abbreviation: VHL, von Hippel-Lindau.

a One patient deteriorated from McCormick grade II to III at 200 months in the context of cumulative VHL-associated tumor burden.

b The patient with sporadic spinal hemangioblastoma had only the first postoperative assessment at our center. This result therefore reflects available postoperative imaging.

## 4 Discussion

In this selected, surgically ascertained referral-center registry, intradural cauda equina sHBLs were documented in 32 of 237 patients, but surgical treatment was infrequent. Eight of 565 procedures (1.4%) involved ten of 804 resected CNS HBLs (1.2%), and eight of the 32 patients (25%) underwent surgery by July 31, 2025, whereas 24 patients had no such procedure recorded at our institution by that date. The majority of patients in our database demonstrated either stable tumor growth or no neurological symptom progression. At our institution, patients were followed by annual whole-neuraxis MRI within the structured VHL surveillance program. However, because the registry was surgically ascertained and the 24 patients with cauda equina sHBLs without resection were not comparatively analyzed, the observed proportion of 8 of 32 patients undergoing resection describes local management only.

Available natural-history data support a cautious and individualized approach to radiographically detected cauda equina sHBLs. In a prospective natural-history study of 225 patients with VHL disease, Lonser et al. identified cauda equina sHBLs in 139 patients (61.8%) at study entry, accounting for 212 of 1921 CNS HBLs (11.0%). Twenty-five percent of cauda equina sHBLs progressed radiologically within 5 years, whereas only five patients (2%) developed attributable symptoms during follow-up, while infratentorial, brainstem and intramedullary lesions progressed in 50% of cases (Lonser et al., 2014). These findings caution against resection based solely on radiographic detection and would lead to numerous unnecessary surgeries (Lonser et al., 2014; Ammerman et al., 2006). In our surgical cohort, the nine VHL-associated tumors had been followed radiographically for a median of 65 months before resection. Surgery was discussed following the development of radicular pain or neurological deficit, or concerning radiographic progression, interpreted in relation to lesion location and the overall clinical context.

The difference between the proportion of cauda equina sHBLs reported by Lonser et al. and that observed in our registry should be interpreted in light of differences in patient selection and imaging protocols. Lonser et al. enrolled patients with VHL with at least one MRI-visible hemangioblastoma, whereas our registry included patients with at least one surgical procedure. Imaging resolution might also have influenced lesion detection: our protocol uses 3 mm sagittal MRI slices as screening (Klingler et al., 2020) whereas Lonser et al. reported 1 mm craniospinal imaging (Lonser et al., 2014).

Cauda equina sHBLs may attain substantial size before producing neurological symptoms. This differs from intramedullary tumors, for which relatively limited growth may cause irreversible myelopathic injury. Nevertheless, radiological progression may become clinically relevant when a cauda equina lesion is located close to the conus medullaris. In one patient in our cohort, a progressively enlarging lesion adjacent to the conus medullaris was resected because further growth was considered to pose a risk of irreversible neurological deficit. This case illustrates why symptoms, radiological behavior, anatomical location, and anticipated neurological risk should be considered together rather than applying a uniform intervention threshold.

Published evidence regarding surgical management for cauda equina sHBLs remains limited, consisting largely of case reports (Fujii et al., 2022; D’Oria et al., 2022), and small surgical series (Han et al., 2024; Mehta et al., 2017). The principal published cauda equina surgical cohorts and the present series are summarized in Table 3. The most directly comparable study is the VHL-associated cohort reported by Mehta et al. (2017). Fifteen patients with VHL disease underwent 18 operations for 21 symptom-producing cauda equina sHBLs, with a median follow-up of 5.9 years. At 6 months, McCormick grade was stable or improved after 17 of 18 operations. Four tumors at three operations were not resected because they originated from motor nerve roots. Among the 15 operations in which resection was attempted, gross-total resection was achieved in 14 and near-total resection in one. One tumor recurred 8 years after intended gross-total resection. In our cohort, all ten tumors were completely resected, McCormick grade was unchanged at the first recorded postoperative follow-up, and no recurrence was identified during a median postoperative imaging follow-up of 67 months. However, Mehta et al. reported a larger VHL-associated surgical cohort and a slightly longer median follow-up. Differences in eligibility criteria, case selection, outcome assessment, and follow-up preclude direct comparison.

**Table 3:** Principal surgical series of cauda equina hemangioblastomas in VHL disease.

Study and cohort	Extent of resection	Functional outcome	Follow-up
Mehta et al., (2017) 15 patients, all VHL 18 operations; 21 HBLs	GTR at 14/15 attempted operations; near-total at 1. Four sHBLs unresected at 3 operations.	McCormick grade stable or improved after 17/18 operations at 6 months.	Median 5.9 years (range: 1.8–13).
Han et al., (2024) 10 patients 6 VHL; 4 sporadic	GTR in 9/10 sHBLs; incomplete resection in 1.	One patient with new weakness and sphincter dysfunction; sphincter deficit persisted at discharge	Mean 103.2 months (range 30–168).
Present study 8 patients 7 VHL; 1 sporadic 8 operations; 10 tumors	Complete resection 10/10 sHBLs	McCormick grade unchanged in 8/8 at first follow-up; radicular pain improved in 6/6.	Median imaging 67 months (range: 11–260); clinical 53 months (range: 3–260).

Differences in eligibility, case mix, assessment timing, and recurrence ascertainment preclude direct comparative estimates.

Abbreviations: GTR, gross-total resection; sHBL, spinal hemangioblastoma; VHL, von Hippel-Lindau.

Han et al. (2024) reported ten surgically treated patients with cauda equina sHBLs, six of whom had VHL disease. Their VHL subgroup generally had greater preoperative neurological impairment, including leg weakness, sensory deficits, sphincter dysfunction, and a modified McCormick grade of III in half of the cases. In contrast, all patients in our surgical cohort had a preoperative McCormick grade of I or II, and none had gait disturbance or bladder dysfunction. The milder preoperative neurological status in our cohort may reflect variations in surveillance strategies, referral patterns, case mix, and surgical decision-making between centers.

Postoperatively, Han et al. reported neurological deterioration with persistent bladder dysfunction in one patient and tumor recurrence requiring repeat surgery in three patients. In our cohort, no deterioration in McCormick grade was observed at the first recorded postoperative follow-up, although one patient developed a cerebrospinal fluid fistula that resolved after lumbar drainage. No recurrence was identified during the available imaging follow-up.

When safely feasible, complete resection is the surgical objective because residual tumor may increase the risk of subsequent progression or recurrence (Wach et al., 2025). Safe microsurgical removal requires identification and interruption of arterial feeders while preserving venous drainage until adequate tumor devascularization has been achieved (Krüger et al., 2019; Samartzis et al., 2016; Takeshima et al., 2023). Intraoperative indocyanine green video angiography may assist in distinguishing arterial feeders from draining veins and in confirming progressive devascularization of the tumor (Klingler et al., 2025). IONM may provide additional information in cauda equina sHBL procedures. Some tumors in our cohort were firmly adherent to individual cauda equina fascicles, and electrical stimulation facilitated identification of motor- and sphincter-related fascicles. These observations suggest that IONM may support intraoperative decision-making in selected cases when functional nerve-root involvement is suspected (Siller et al., 2017). IONM was used selectively in three of eight procedures at the operating surgeon's discretion, with greater use in the later years of the series. Its use in only three procedures does not permit conclusions regarding its effect on neurological outcome.

Because HBLs are highly vascular, preoperative embolization has been proposed as a means of reducing intraoperative blood loss and facilitating tumor dissection (Ampie et al., 2016a, 2016b). The largest tumor in our cohort (2078 mm^3^) underwent preoperative partial particle embolization with estimated devascularization of 40–50%. Intraoperative EBL was 200 mL, compared with our cohort median of 170 mL. This single case does not establish benefit. This case was part of a larger embolization feasibility study at our clinic (Doria-Medina et al., 2026). Embolization may be considered in selected cases after weighing the anticipates benefit against the additional procedural risks (Ampie et al., 2016a, 2016b; Haruma et al., 2025; Doria-Medina et al., 2026).

Belzutifan, an oral hypoxia-inducible factor 2α inhibitor, represents an emerging systemic treatment option in VHL disease (Rhein et al., 2025). Though none of the patients in our cohort received systemic therapy, belzutifan could become relevant for selected patients with multiple or progressive cauda equina sHBLs, particularly when repeated surgery would result in substantial cumulative morbidity or when other VHL-associated tumors also require treatment. However, evidence specific to cauda equina sHBLs is lacking, and systemic therapy should not be regarded as a substitute for timely surgical decompression in patients with progressive neurological deficits or lesions requiring intervention.

In summary, our findings suggest that resection of selected intradural cauda equina sHBLs can preserve functional status, improve radicular pain, and provide durable local control. They do not establish the comparative safety of surveillance or define an evidence-based threshold for surgery. Management should therefore remain individualized according to symptoms, radiological progression, lesion location, functional nerve-root involvement, cumulative VHL-associated disease burden, and the broader clinical context.

## 5 Limitations

This study has several limitations. Its retrospective design and surgical ascertainment introduce potential selection and information bias, and the small cohort limits generalizability. Treatment decisions were individualized, and the available data do not permit standardized adjudication of surgical necessity. The characteristics and outcomes of the 24 patients without cauda equina resection were not comparatively analyzed; therefore, no inference regarding the safety or superiority of surveillance versus surgery is possible. Although widely used, the McCormick scale may not adequately capture subtle neurological deficits, radicular pain, or patient-reported outcomes specific to cauda equina lesions. Finally, the burden and progression of other VHL-associated manifestations were not systematically accounted for.

Centralized prospective data collection and extended postoperative long-term follow-up provide useful descriptive observations for this rare tumor location. Our study provides several insights, supported by consistent surgical technique, standardized outcome assessment, and long-term follow-up with centralized prospective data collection. The findings should be regarded as hypothesis-generating and may inform future multi-institutional studies and prospective registries incorporating standardized follow-up of both operated and non-operated lesions.

## 6 Conclusions

Within this selected, surgically ascertained single-center VHL/HBL registry, intradural cauda equina sHBLs were documented in 32 patients and resected in eight. Ten tumors were removed for symptoms and/or radiological progression concomitantly during surgery for another lesion, if irreversible neurological damage could be considered imminent (i.e. at the conus medullaris), or for diagnostic clarification. Complete resection was achieved in all ten tumors, functional status was preserved short-term, and no tumor recurrence was observed after long-term surveillance. These observations support an individualized surgical strategy incorporating symptoms, anatomical risk, and radiological progression with continued MRI surveillance at a dedicated VHL center. The study does not establish the comparative safety of surveillance or define an evidence-based threshold for intervention. Multicenter studies and prospective registries are needed to refine surgical indications and optimize long-term management.

## Author contributions

All authors contributed to the study conception and design. Material preparation, data collection and analysis were performed by Roberto Doria-Medina, Christine Julia Gizaw, Jan-Helge Klingler, Marc Hohenhaus, Romina Hohenhaus, Nicolas Neidert, Kilian Rhein, and Athina Ganner. Supervision and resources: Roberto Doria-Medina, Jan-Helge Klingler and Jürgen Beck. The first draft of the manuscript was written by Roberto Doria-Medina and Jan-Helge Klingler and all authors commented on previous versions of the manuscript. All authors read and approved the final manuscript.

## Ethical statement

This study was performed in line with the principles of the Declaration of Helsinki. Approval was granted by the Ethics Committee of University of Freiburg (No. 79/20). Informed consent was obtained from all individual participants for inclusion in the VHL/HBL registry.

## Funding

We acknowledge support by the Open Access Publication Fund of the 10.13039/501100002714University of Freiburg.

## Declaration of competing interest

The authors declare that they have no known competing financial interests or personal relationships that could have appeared to influence the work reported in this paper.
