# Supplementary material for: Intradural cauda equina hemangioblastomas: Surgical management and long-term outcomes at a von Hippel–Lindau referral center
Source: Brain Spine. 2026 Oct 12;6:107479. doi: 10.1016/j.bas.2026.107479 (PMC13694938; doi:10.1016/j.bas.2026.107479)
Supplement: Supplementary file 1 [file mmc1.docx]

| **Supplementary Figure 1.** Representative case of an intradural cauda equina hemangioblastoma in a patient with VHL disease. | |
| --- | --- |
|  |  |
|  |  |
| **Supplementary Figure 1.** Yearly T1 weighted MRI imaging with contrast enhancement of the CNS axis is performed as the standard of care in patients with VHL disease. This is a representative case of an intradural cauda equina sHBL in a female patient with VHL disease. The patient had been diagnosed with VHL disease at the age of 4 years. (A) At 19 years of age, surveillance MRI revealed a first manifestation of an intradural cauda equina sHBL involving the right L3 nerve root. As the lesion was asymptomatic, the patient was monitored with annual clinical and radiological follow-ups. (B and C) At 25 years of age, the patient developed progressive radicular pain, numbness, and paresis corresponding to the affected L3 nerve root. Because of tumor progression and tumor-attributable symptoms, surgical resection was recommended. Due to neuroforaminal tumor extension and vascular supply, preoperative embolization was performed two days before surgery, achieving partial devascularization. After interlaminar fenestration through a partial laminectomy of L3 and L4, intraoperative neuromonitoring was used to identify motoric nerve-root fascicles. Complete resection of the sHBL was achieved. Postoperatively, radicular pain and paresis resolved completely, while numbness partially improved. (D) At last postoperative MRI follow-up, no tumor recurrence was observed.  Abbreviations: CNS, central nervous system; MRI, magnetic resonance imaging; sHBL, spinal hemangioblastoma; VHL, von Hippel-Lindau | |
