# Supplementary material for: Intradural cauda equina hemangioblastomas: Surgical management and long-term outcomes at a von Hippel–Lindau referral center
Source: Brain Spine. 2026 Oct 12;6:107479. doi: 10.1016/j.bas.2026.107479 (PMC13694938; doi:10.1016/j.bas.2026.107479)
Supplement: Supplementary file 2 [file mmc2.docx]

| **Supplementary Table 1.** Operative characteristics of eight procedures in eight patients involving ten intradural cauda equina hemangioblastomas | |
| --- | --- |
| **Procedure-level characteristics (n = 8 procedure)** |  |
| Operative duration, median (range), min | 228 (147-400) |
| Estimated blood loss, median (range), mL | 170 (100-500) |
| Intraoperative blood transfusion, n (%) | 0 (0.0) |
| Intraoperative neuromonitoring, n (%) | 3 (37.5) |
| Preoperative embolization, n (%) | 1 (12.5) |
| **Number of tumors resected per procedure, n (%)** |  |
| One tumor | 6 (75.0) |
| Two tumors | 2 (25.0) |
| **Exposure level characteristics (n = 9 exposures)** |  |
| **Surgical approach, n (%)** |  |
| Hemilaminectomy | 4 (44.4) |
| Laminotomy | 3 (33.3) |
| Partial hemilaminectomy/Hemilaminotomy | 1 (11.1) |
| Facetectomy | 1 (11.1) |
| **Retractor type, n (%)** |  |
| Traditional retractor | 8 (88.9) |
| Tubular retractor (minimally invasive) | 1 (11.1) |
| **Dural closure, n (%)** |  |
| Sutures alone | 1 (11.1) |
| Sutures with augmentation | 8 (88.9) |
| Augmentation material among augmented closures (n = 8), n (%) |  |
| TachoSil® alone | 3 (37.5) |
| Gelita® alone | 1 (12.5) |
| Fibrin glue alone | 1 (12.5) |
| Fibrin glue + TachoSil® | 1 (12.5) |
| Fibrin glue + Gelita® | 1 (12.5) |
| Fibrin glue + TachoSil® + autologous muscle tissue | 1 (12.5) |
| Data are presented as n (%) unless otherwise stated. One procedure required separate exposures at two spinal levels, resulting in nine exposures across eight procedures. | |
